# The NuroSleeve, a user-centered 3D printed hybrid orthosis for individuals with upper extremity impairment

**DOI:** 10.1186/s12984-023-01228-2

**Published:** 2023-08-04

**Authors:** Mehdi Khantan, Mikael Avery, Phyo Thuta Aung, Rachel M. Zarin, Emma Hammelef, Nabila Shawki, Mijail Demian Serruya, Alessandro Napoli

**Affiliations:** 1 Raphael Center for Neurorestoration, Thomas Jefferson University, Philadelphia, PA 19107 USA; 2 Department of Electrical and Computer Engineering, Temple University, Philadelphia, PA 19121 USA; 3 Studio Krea, Collingswood, NJ 08108 USA

**Keywords:** Assistive, Exoskeleton, Hand therapy, Rehabilitation, Stroke, Wearable robotics, Three dimensional (3D) printed active orthoses, Upper extremity impairment

## Abstract

**Background:**

Active upper extremity (UE) assistive devices have the potential to restore independent functional movement in individuals with UE impairment due to neuromuscular diseases or injury-induced chronic weakness. Academically fabricated UE assistive devices are not usually optimized for activities of daily living (ADLs), whereas commercially available alternatives tend to lack flexibility in control and activation methods. Both options are typically difficult to don and doff and may be uncomfortable for extensive daily use due to their lack of personalization. To overcome these limitations, we have designed, developed, and clinically evaluated the NuroSleeve, an innovative user-centered UE hybrid orthosis.

**Methods:**

This study introduces the design, implementation, and clinical evaluation of the NuroSleeve, a user-centered hybrid device that incorporates a lightweight, easy to don and doff 3D-printed motorized UE orthosis and a functional electrical stimulation (FES) component. Our primary goals are to develop a customized hybrid device that individuals with UE neuromuscular impairment can use to perform ADLs and to evaluate the benefits of incorporating the device into occupational therapy sessions. The trial is designed as a prospective, open-label, single-cohort feasibility study of eight-week sessions combined with at-home use of the device and implements an iterative device design process where feedback from participants and therapists informs design improvement cycles.

**Results:**

All participants learned how to independently don, doff, and use the NuroSleeve in ADLs, both in clinical therapy and in their home environments. All participants showed improvements in their Canadian Occupational Performance Measure (COPM), which was the primary clinical trial outcome measure. Furthermore, participants and therapists provided valuable feedback to guide further development.

**Conclusions:**

Our results from non-clinical testing and clinical evaluation demonstrate that the NuroSleeve has met feasibility and safety goals and effectively improved independent voluntary function during ADLs. The study’s encouraging preliminary findings indicate that the NuroSleeve has met its technical and clinical objectives while improving upon the limitations of the existing UE orthoses owing to its personalized and flexible approach to hardware and firmware design.

*Trial Registration:* ClinicalTrials.gov identifier: NCT04798378, https://clinicaltrials.gov/ct2/show/NCT04798378, date of registration: March 15, 2021.

## Background

Neuromuscular disorders impose a significant socioeconomic burden on society. There are over 7 million stroke survivors in the United States alone [1, 2], 62% of whom have a loss of dexterity in their upper extremities (UE) [3]. Approximately 291,000 Americans are living with disability due to spinal cord injury (SCI) [4], and Duchenne Muscular Dystrophy (DMD) and Becker Muscular Dystrophy (BMD) combined affect around 14 in 100,000 American males [5]. Neurological disorders and diseases often result in permanent disability that prevents individuals from performing Activities of Daily Living (ADLs) independently [4, 6]. Stroke [1, 7, 8], SCI [4, 9, 10], and muscular dystrophy (MD) frequently result in debilitating UE motor impairments that persist beyond rehabilitation discharge [11, 12]. Individuals with moderate to severe neurological UE impairment frequently exhibit limited active movement in their paretic elbow and little to no active movement in their paretic wrists and fingers [13, 14].

Rehabilitation therapies that implement assistive neurotechnology devices tend to improve functional motor recovery, reducing impairment and improving independence in ADLs, quality of life, and community participation [15–19]. Over the past six decades, it has been shown that the use of active wearable neurotechnology devices benefits individuals living with UE impairment by helping them perform ADLs [20–22].

Currently, commercially available active UE orthoses for home use can be divided into two groups: (1) mechanically actuated orthoses [23–27], which use either electric or pneumatic motors to achieve motion; and (2) Functional Electrical Stimulation (FES) orthoses [28–30] which electrically stimulate muscles to achieve motion. The application of electrical stimulation to a person's muscles depolarizes peripheral neurons and elicits muscle contractions, allowing the individial to perform a movement. FES can benefit individuals by substituting or enhancing movement. Repeated muscle activation using FES may also increase voluntary motor control. This suggests that the use of FES devices improves motor recovery and can serve as a rehabilitation technique as well as assist with ADLs [31, 32]. Thus, FES has evolved into a crucial treatment approach that clinicians may use to help individuals with stroke and SCI regain the capacity to stand, walk, reach, and grasp [33].

Widespread use of the cummercially available active orthoses is hindered by several factors: (1) the challenge of making them form-fitting, comfortable, authentic, easy to use, portable, and lightweight; (2) the inability to customize the placement of sensors as input controls and effectors to optimize user movements; and (3) the lack of rehabilitation professionals skilled in training individuals on how to integrate the orthosis into daily routines. To the best of our knowledge, no currently available commercial orthosis offers all the aforementioned factors for restoring UE functionality during ADLs in “real-world” situations [34–41].

Academically fabricated motorized UE orthoses have their own limitations; they often need to be fixed to a wheelchair or stationary surface (e.g., a table) [42–44] or require support from the person’s back and shoulders [45] in order to function. Our clinical experience suggests that most individuals would not find such devices practical to use in ADLs or in the community. Soft robotic sleeves [46–52] provide an alternative to motor-based approaches; however, such sleeves are not easy to don and doff, and most require an air compressor or compressed gas tanks to function. Hence, soft robotic sleeves may not easily find their market without first addressing their practicality and usability issues.

To overcome the limitations of the currently available UE orthoses, the next generation of UE devices must be simple to use to encourage acceptance and integration in ADLs while having favorable aesthetics for widespread adoption. Specifically, a user-friendly orthosis should be effective, comfortable, portable, form-fitting, safe and easy to use, easy to don and doff, and offer the individual a variety of options for controlling it. Furthermore, it should be lightweight; this is paramount because continuous usage of heavy UE orthoses may have a detrimental effect on user satisfaction and compliance and may contribute to physical problems such as pressure point formation, muscular fatigue, perspiration, and skin irritation. Also, an important requirement for the UE orthoses to be effectively incorporated into ADLs may be the battery life of more than 8 h, making it possible to be used each day on a single battery charge. These numerous requirements cannot be met in devices that are designed following the “one-size-fits-all” principle without accounting for the unique needs of each individual [34–41].

This study introduces the design, development, implementation, and clinical evaluation of the NuroSleeve, a novel user-centric active UE orthosis. The NuroSleeve design accommodates the unique needs and conditions of individuals by integrating (1) a user-specific control mechanism, (2) a custom 3D-printed, lightweight, and easy to don and doff motorized splint, and (3) an off-the-shelf FES unit to take advantage of the electrical stimulation benefits [34–37, 40]. In addition, the design process incorporates feedback from individuals to tailor the system components and functionality for their personal needs and inform overall design improvements. Our goal in developing the NuroSleeve is to meet the unique needs of individuals and promote the adoption of the technology in clinical settings, at home, and in the community.

## Methods

**Fig. 1:**
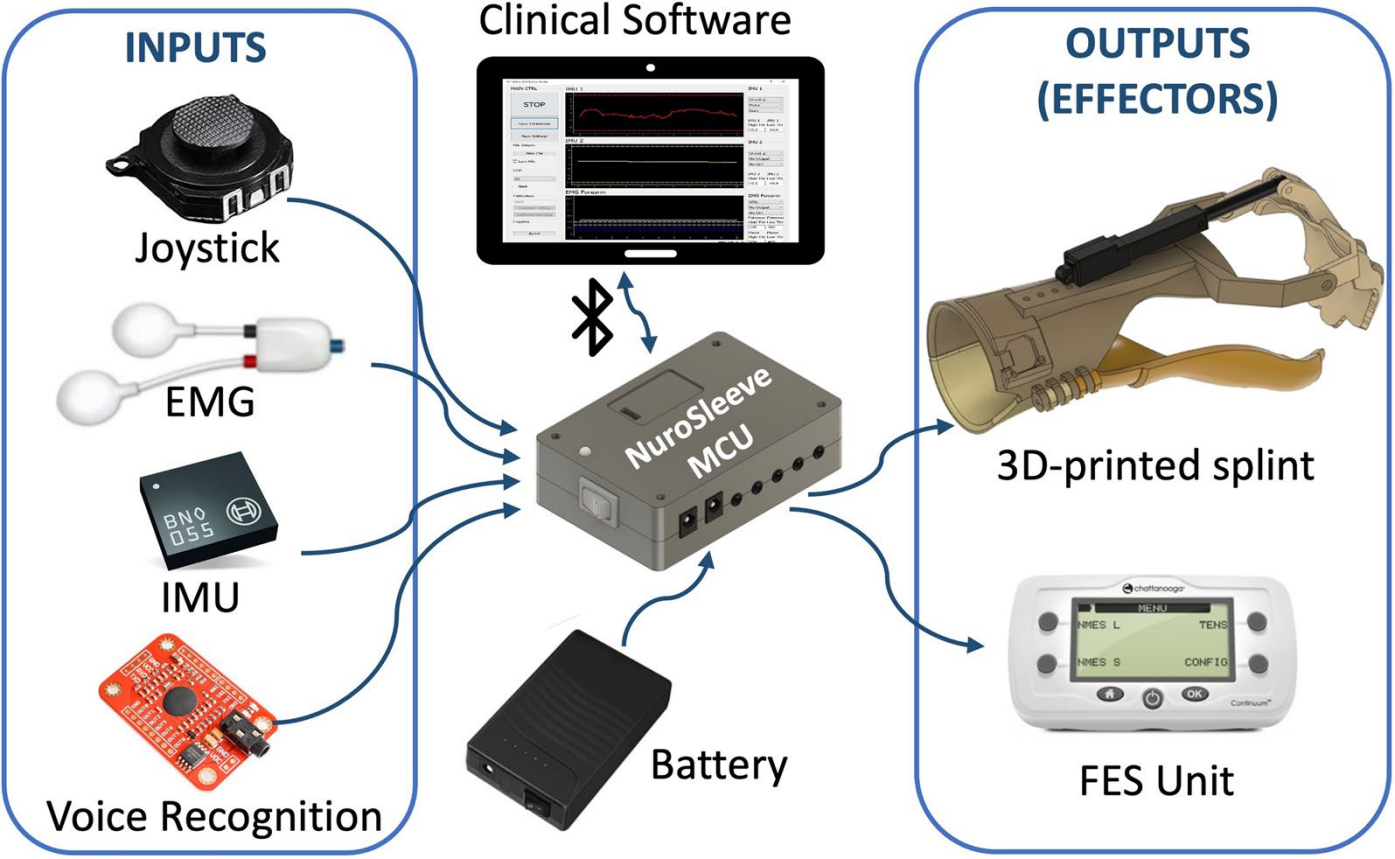
The NuroSleeve consists of the main control unit (center), which accepts different input control signals (left) to control one or more end effectors (right). Implementations are customized for each patient by the occupational therapist using the clinical configuration software

The NuroSleeve comprises six main components: a custom 3D-printed splint, an external FES unit, a main control unit (MCU), a clinical software suite for configuration, a rechargeable battery and a series of input sensors, as shown in Fig. 1. The custom firmware running on the MCU receives signals from one or more input sensors, processes the data in real time, and derives control signals for the effectors. The NuroSleeve firmware and hardware allow for user-specific sensor setup and a personalized input and output mapping. In other words, sensor placement and user inputs are customized for the user, as are the effectors and corresponding control strategies. The NuroSleeve can be controlled by one or more of the following control inputs: joystick input, electromyography (EMG) signals, inertial measurement unit (IMU) signals, and voice control. Current effector options include a motorized 3D-printed forearm splint and an external two-channel clinical-grade FES device (see Fig. 1).

It is important to emphasize that the NuroSleeve device is user-specific and is personalized with the help of an occupational therapist (OT) using the clinical configuration software. The OT can exploit rehabilitation therapy principles to help identify the optimal combination of sensor inputs and effectors and the optimal placement of each (including FES electrodes) based on the individual’s unique abilities, needs, and functional goals. The OT also elicits feedback from the user about what configuration of the device and control mechanism may be more intuitive or easier to use. This information is relayed to the design team, which then designs and implements the solution for the participant, and/or uses the feedback to inform improvements to the overall system design. Via wireless Bluetooth connectivity, the clinical software suite allows for device setup, configuration, usage monitoring, real-time data collection and visualization.

The NuroSleeve splint is customized for each individual using 3D scanning and printing technologies. Each splint is built to accommodate the unique anatomy and impairment of the user to maximize comfort, efficacy, and fit. We refer to this customization process as the “digital orthotist” process, as it combines modern industrial design techniques with occupational therapy and orthotics know-how. The process starts with a 3D scan of the user’s impaired hand and forearm, which is then used to build a custom computer-aided design (CAD) model of the splint. The model may be fine-tuned to suit the user as necessary, and then it is 3D printed. The combination of 3D scanning and printing technologies together with user feedback integration has facilitated our development of an orthosis that is lightweight, aesthetically pleasing, and form-fitting in key locations while form-adjusted in others to avoid pressure points and bony prominences [41]. The splint has an innovative clamshell design, incorporating a hinge on one side that allows the thumb and cuff sections to be opened and closed, maximizing the individual’s ability to don and doff without assistance. The use of 3D-printed rigid plastic components rather than fabric or other soft materials, provides mechanical support and enables dynamic grasp properties, which are particularly relevant for individuals with spasticity (e.g., excessive tone) [53]. The NuroSleeve also integrates an external FES unit that can generate functional movement by directly stimulating muscles. Therefore, while NuroSleeve is intended to be used as an assistive device, it can also be used as a self-modulating rehabilitation device.

### Device control method

The NuroSleeve can control the linear actuator of the splint and/or the FES unit using any of the following control modes: (1) Manual control. A small joystick is fitted to the device at an accessible location for the user, either attached to the splint or as a separate handheld device; (2) Voice Activation. A voice recognition module that does not require internet connectivity (this was added to the system following user feedback) is incorporated into the MCU. This module uses software that learns voice commands and maps specific spoken phrases onto actions (e.g., “open”, “close”, “stimulate”) during a single calibration session. The voice recognition training takes a few minutes for each voice command. After training, the parameters are stored on the module and the individual can use the voice control option to control the NuroSleeve independently without the use of cloud connectivity; (3) EMG control. The device’s two EMG channels can be set up to control the linear actuator and/or FES with a multi-threshold approach, in which one or two signal thresholds are set up to trigger the effectors. The threshold values and their use in controlling the effectors can be customized via the clinical software suite; (4) IMU control. The NuroSleeve can leverage up to two IMUs for splint and/or FES control. The IMU sensors may be attached to a clip, placed in a band or other fastener and positioned in user-selected places (e.g., shoelaces, contralateral wrist, eyeglasses). In IMU mode, the device can be operated in two different configurations: continuous or discrete. Continuous control configuration uses a multi-threshold approach and makes use of the IMU's 3D orientation data to continuously control the linear actuator and/or FES effectors. The discrete control configuration uses a tap-and-go control approach, in which the system uses the IMU 3D acceleration data to fully open or close the hand; each tap on the IMU sensor toggles an ON/OFF actuation. In other words, the IMU sensors can function as a toggle switch to control a two-state machine based on the status of the effector.

### The splint

**Fig. 2:**
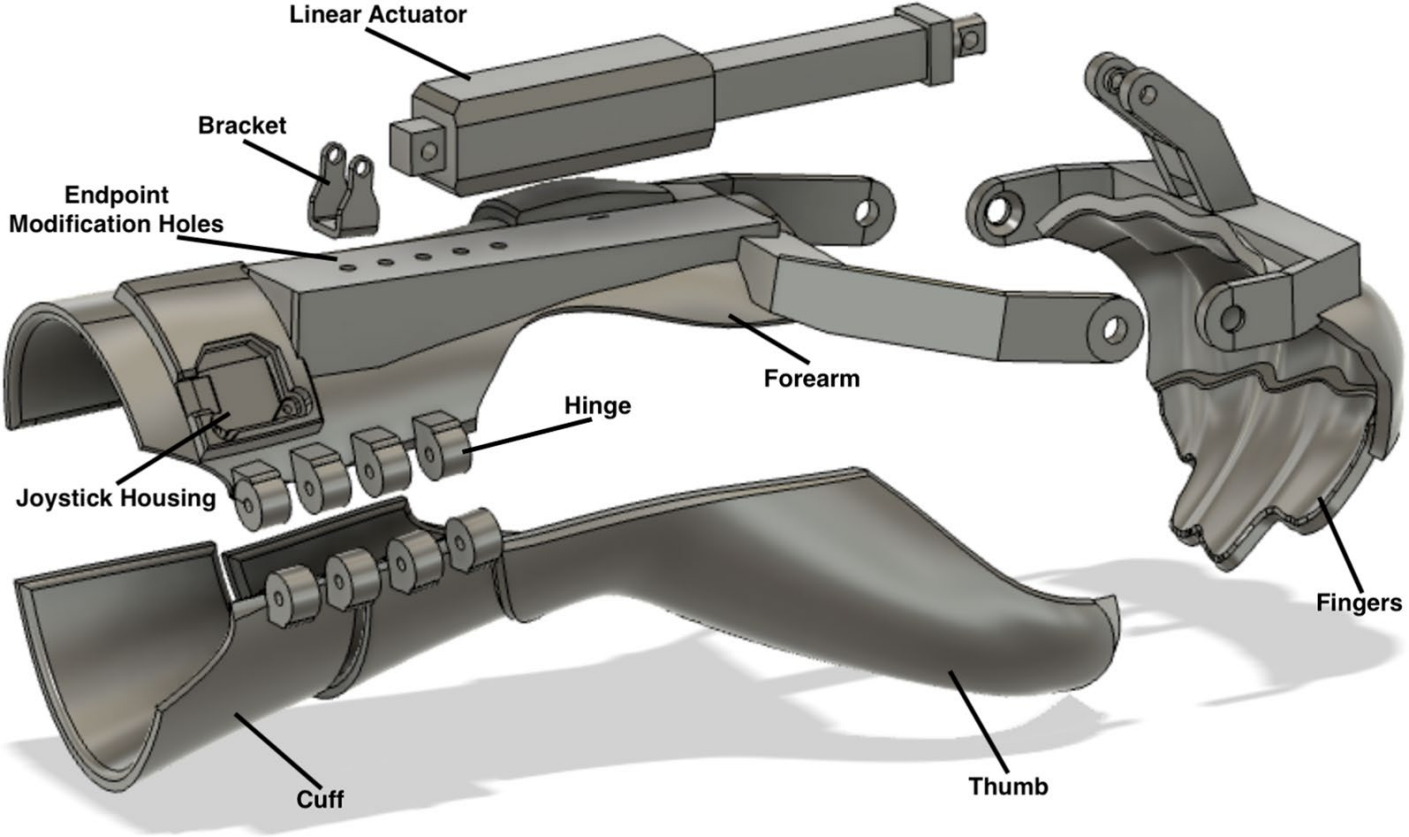
Exploded view of NuroSleeve splint components, including thumb, fingers, arm, and cuff sections

The NuroSleeve 3D printed custom splint consists of four main sections: forearm, cuff, thumb, and fingers, as shown in Fig. 2. The fingers section facilitates the opening and closing of the hand and is assisted by a splint-mounted, low-profile, lightweight electro-mechanical linear actuator (PA-07, Progressive Automations, Arlington, WA) [54]. The linear actuator has a 50-mm stroke and an integrated current limiting circuit as a safeguard mechanism to avoid overtravel. The stationary part of the linear actuator is connected to the forearm section, while the actuated rod is connected to the finger section (see Fig. 2). When the linear actuator rod is extended forward, the finger piece assists the user with grasping, when the rod is retracted, it assists with hand opening. This allows the user to achieve functional flexion and extension of the metacarpophalangeal (MCP) joint of the affected hand. The forearm section of the splint has multiple endpoint modification holes for mounting the linear actuator so that its position can be adjusted. This allows for changing the start and end positions of the MCP joint’s flexion and extension while keeping the range of motion (ROM) fixed. This approach allows the OT to customize the individual’s ROM endpoints, based on their clinical conditions and functional needs. Moving the linear actuator distally along the forearm, for instance, enables the user to grasp smaller objects, whereas mounting the linear actuator proximally can facilitate the grasping of larger objects. As part of the NuroSleeve calibration process, safe and optimal hand motion and grasp are carefully verified and validated by an OT to avoid any potential for injuries, such as repeated hyperflexion or hyperextension of fingers and soft tissue injuries [55].

### Digital orthotist process

**Fig. 3:**
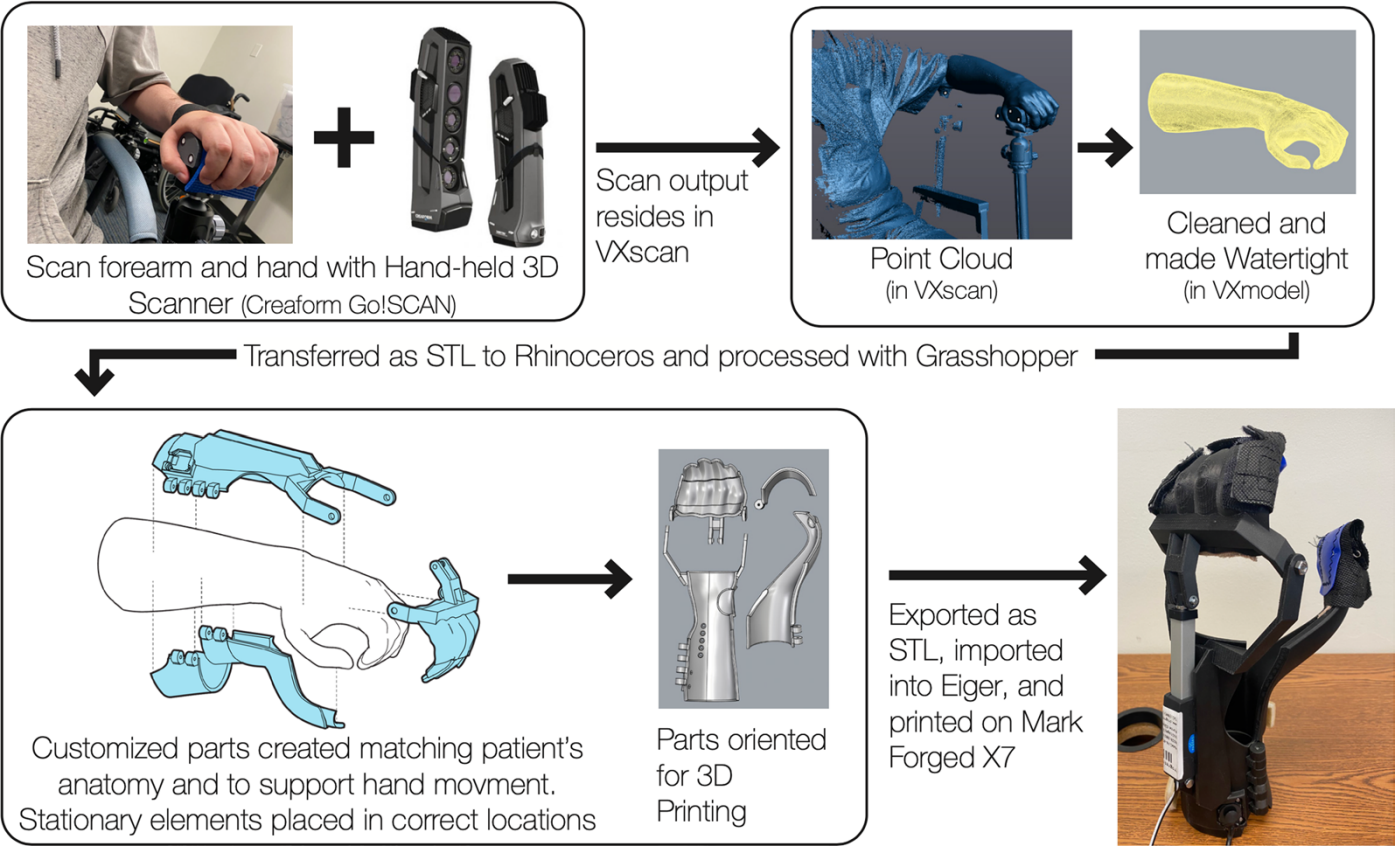
The “digital orthotist" process begins with a 3D scan of the individual's hand and forearm. The resulting 3D mesh is then processed and imported into Rhinoceros, where, with the help of custom Grasshopper scripts, is converted into a personalized 3D-printable splint. Finally, the 3D-printed splint is built and assembled

To create a user-centric device that effectively matches the user's hand anatomy and functional requirements, we devised a digital splint design process that combines modern industrial design and occupational therapy techniques. This “digital orthotist” process (as shown in Fig. 3) begins with a 3D scan of the subject’s forearm and hand using the Creaform Go!SCAN 3D scanner [56], which features a volumetric accuracy [57] of 0.050 mm ± 0.150 mm/m [58] and uses proprietary software (VXmodel) [59] to create a watertight model by removing scan artifacts and superfluous information (e.g., chest-related data), overlapping or coarse surfaces, and holes. Once created, the individual watertight model is then imported into Rhinoceros [60], a 3D CAD software that features powerful design and modeling tools ideal for the creation of the custom 3D-printable UE splint. To improve the reliability and repeatability of the "digital orthotist” process, automation scripts are created within Grasshopper [61], a parametric programming tool and native plugin of Rhinoceros. While some common design elements, such as the hinge, endpoint modification holes, and joystick housing, can be rescaled and modified for use in multiple models, the profiles of the forearm, cuff, thumb, and finger sections of each model are unique, custom-designed by processing the user’s UE 3D scan data.

Once the splint customization and creation have been completed, the 3D splint files are printed on a Markforged X7 industrial 3D printer [62] using Onyx™ microcarbon fiber-filled nylon [63]. Compared to other 3D printing materials, such as Nylon or Acrylonitrile Butadiene Styrene (ABS), Onyx™ produces orthoses with superior chemical resistance, rigidity, and flexural stress [64, 65]. All sections of the splint are printed such that the skin-contact surfaces are face up and thus not in contact with the build’s support scaffold. This ensures that the skin-contact surfaces are as smooth as possible to reduce skin irritation. Printing a complete NuroSleeve splint requires approximately 48 h.

After printing and assembly of the splint, fabric and straps are added at specific locations to secure the user’s forearm, hand, and fingers in place. Namely, custom Oly Fun fabric wraps are attached to the finger piece and thumb piece to create a mitten-like pocket for the fingers, Oly Fun fabric was chosen because it is non-stretchy, allowing the fingers to stay open despite resistance, and it is non-woven, so it does not fray like other fabrics. It also breathes well, which reduces sweating and the likelihood of skin irritation. Finally, Rolyan straps and hook-and-loop tapes are used to secure the cuff and thumb sections to the forearm, preventing the clamshell hinge from opening. Rolyan straps, which are recognized for their softness and flexibility, were chosen to reduce the risk of causing skin irritation.

### The functional electrical stimulation component

As depicted in Fig. 1, the NuroSleeve incorporates a two-channel commercially available neuromuscular stimulator, the Chattanooga Continuum [66]. This FES device is intended for use in rehabilitation to alleviate pain with Transcutaneous Electrical Nerve Stimulation (TENS) and cure muscle weakness with Neuromuscular Electrical Stimulation (NMES). This integration enables the Neurosleeve to control FES stimulation via its multiple control methods, thereby transforming the device into a FES orthosis. The stimulation can be activated by any of the input control signals of the NuroSleeve. The individual does not need to don 3D-printed orthoses in order to utilize the FES system; instead, they can simply attach the provided electodes to the intended muscles. Electrode placement is determined based on the clinical judgment of the occupational or physical therapist working with the participant and the desired functional outcome. Once the target muscle is identified, it usually only takes a few minutes for the therapist to identify and verify the optimal placement. The therapist can then instruct the participant and caregivers on how to use anatomical landmarks to place the sticker electrodes. During the trial, the therapist confirms the participant is capable of placing the electrodes in the correct location by observing them, both in person and then subsequently via video teleconference.

The FES device is configured to generate galvanically isolated, biphasic, charge-balanced, and current-controlled pulses with a frequency of 35 Hz and a pulse width of 300 µs. The stimualtion intensity (current amplitude) is selected and set for each individual by an OT when setting up and calibrating the device. To determine the appropriate FES intensity for an individual, the OT begins stimulating the targeted muscle at a low intensity and progressively increases the intensity until a level of muscle contraction sufficient for a functional movement is achieved without causing discomfort. This intensity is proportional to the individual's body structure and the targeted muscle and can reach up to 100 mA (amplitudes beyond 50 mA are not tested if no discernible muscle contraction is elicited.). It is worth noting that while the beneficial effects of FES rehabilitation devices on spasticity are well known, for participants with high levels of spasticity, the use of FES alone may not be enough to achieve functional UE motion, or may require stimulation intensities that are intolerable. This was the sole rationale for developing a hybrid orthosis that combines a motorized orthosis with a FES orthosis to give the OT more options to choose from based on the severity of the injury and the rehabilitation stages.

### The NuroSleeve main control unit

**Fig. 4:**
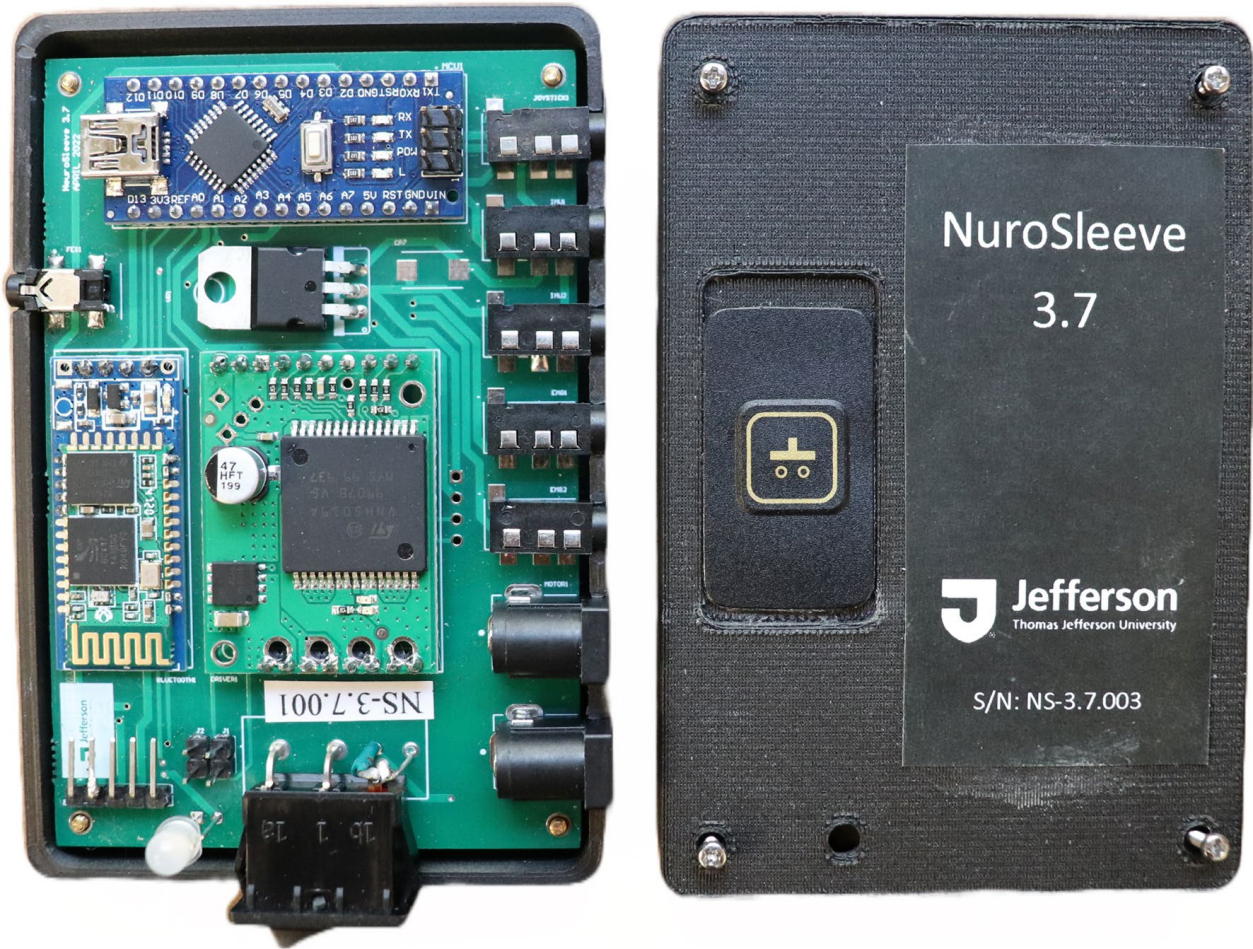
Main Control Unit (MCU) of the NuroSleeve with the 3D printed housing. Each MCU is assembled and tested in house before being deployed in the clinical trial

The NuroSleeve MCU has been designed, assembled, and evaluated in-house at the Raphael Center for Neurorestoration of Thomas Jefferson University and integrates all of its required electronic components (as shown in Fig. 4). To minimize electromagnetic interference (EMI) from other devices, the NuroSleeve's printed circuit board (PCB) was designed according to industry-standard best practices, with adequate isolation and spacing for ground, power, analog, and digital trace signals. This will ensure the safety of the user in the presence of other medical devices, such as muscle stimulators or other devices that can generate additional electromagnetic noise.

The MCU enables control of the effectors by the various control sensors. Its core component is an Arduino Nano controller module [67] with an ATMega328p [68] microcontroller, which runs the NuroSleeve firmware and allows for data collection from up to two wired Bosch BNO055 [69] intelligent 9-axis IMUs (for motion control), up to two MyoWare 2.0 Muscle Sensors [70] (for EMG control), an ELECHOUSE Voice Recognition Module V3 [71] (for voice control), and/or a small joystick controller (for manual control). A description of the device control logic and operating principles is provided in the section below titled "Multi-threshold Control Approach".

Bluetooth connectivity has been implemented via an onboard DSD TECH HC-05 [72] Bluetooth module. Additionally, the NuroSleeve proprietary Bluetooth communication protocol allows easy integration with third-party applications. The NuroSleeve device (including the linear actuator and the MCU) is powered by a single TalentCell 12 V, 3000 mAh Lithium-ion (Li-ion) battery pack [73], consisting of three 18,650 Li-ion batteries in series. To ensure adequate electrical safety, the battery pack has an integrated circuit for protection against overcharging, overdischarging, and short circuits.

### Software suite

The Python-based software suite has been developed in-house to provide real-time management and configuration of the NuroSleeve system via Bluetooth. The software features two separate versions: a Clinical Software Suite and a User Software Suite. The Clinical Suite is designed to be used by a trained therapist and can be used to: (1) customize input and output mapping; (2) adjust sensitivity and use of the input sensors; (3) visualize sensor data and settings in real-time; and (4) access and store the user compliance data from the MCU. The user suite is intended for home usage by individuals and their caregivers; it has less functionality than the Clinical Suite but permits the user to adjust the sensitivity of the sensors.

### User compliance data monitoring

The NuroSleeve is capable of logging user compliance data and storing it onboard on a dedicated flash memory chip. This feature allows therapists and clinicians to monitor at-home device use between clinical appointments enabling Remote Therapeutic Monitoring (RTM), for which the U.S. Centers for Medicare and Medicaid Services (CMS) recently established payment policies [74]. The monitored compliance data can include the total time of device use, the amount of time spent opening and closing the hand, and the time spent in each operation mode (i.e., joystick, voice, IMU, EMG), since the clinician’s last reset. These data can help inform therapy goals and outcomes.

### Multi-threshold control approach

The control strategy of the NuroSleeve can be customized to the individual's physiological needs and preferences through a quick calibration and setup phase integrated into the clinical software suite. The primary customization involves the mapping between selected input control signals (sensors) and desired outputs (effectors). Together, the individual and the treating therapist can determine which of the available inputs is most effective in controlling the effectors for that individual. If IMU inputs are selected, the sensors can be configured for continuous or toggle control. From there, the therapist and the individual can select the optimal sensor placement and activation movement for effector control. The BNO055 IMU is a System in Package (SiP) that integrates a tri-axial 14-bit accelerometer, a tri-axial 16-bit gyroscope, a tri-axial geomagnetic sensor, and an ARM cortex M0 + microcontroller that executes Bosch Sensortec sensor fusion software. As outputs of the IMU intrinsic sensor fusion algorithm, the NuroSleeve MCU reads 3D acceleration and 3D angular displacement in Euler angles via I2C interfaces. The use of fusion technology integrates data from the three aforementioned sensors and eliminates drift (the progressive accumulation of error over time), which makes the sensors more accurate and reliable.

For continuous control, the effectors are controlled by crossing one of two custom-determined thresholds (higher and lower) of the selected input signal. In this mode, one of the three fusion-derived Euler angle data points of the IMU is selected as the input signal (Sig(t)), depending on the IMU's placement. This placement of the IMU is determined by the OT based on the individual's abilities, needs, and functional objectives. A positive crossing of a higher threshold triggers an effector command, while a negative crossing of a lower threshold triggers the opposite command. When the signals fall between two thresholds, the effector maintains its current state. The following equations describe how a selected sensor channel can determine the output command to a selected effector.

$$\mathrm{Effector \, Command}\left(t\right)=\left\{\begin{array}{l}OPEN, \, if\left(Sig\left(t\right)>HighThr\right)\\ CLOSE, \, if\left(Sig\left(t\right)<LowThr\right)\\ HOLD, \, if\left(LowThr\le Sig\left(t\right)\le HighThr\right),\end{array}\right.$$

where:

- $\mathrm{Sig}\left(t\right)$ is the current input signal channel (sensor) value at time t
- $\mathrm{HighThr}$ is a fixed higher threshold value configured with the software
- $\mathrm{LowThr}$ is a fixed lower threshold value configured with the software
- $\mathrm{OPEN}, \mathrm{CLOSE} \mathrm{and} \mathrm{HOLD}$ are the possible output commands at time t

If discrete control is selected, the NuroSleeve’s effectors are controlled with the IMU sensor acting as a toggle switch. In this configuration, the IMU signal of interest is acceleration. The IMU also provides fusion-derived absolute acceleration in three axes at time t (a_x_(t), a_y_(t), and a_z_(t)); the total relative acceleration can be calculated by subtracting gravitational acceleration from the square root of the sum of squared accelerations in three axes. This relative acceleration is eventually thresholded and employed to change the state of the effector (OPEN or CLOSE for the motor, ON or OFF for the FES). This setup is ideal when the user desires to trigger the opening or closing of their hand by tapping the IMU sensor.

$$sig\left(t\right)=\sqrt{{a}_{x}^{2}\left(t\right)+{a}_{y}^{2}\left(t\right)+{a}_{z}^{2}\left(t\right)}-9.8$$

$$Effector \, Command\left(t\right)=\left\{\begin{array}{l}FULLY \, OPEN, \, if\left(Status==CLOSED \, AND \, Sig\left(t\right)>Thr\right) \\ FULLY \, CLOSE, if\left(Status==OPEN \, AND \, Sig\left(t\right)>Thr\right)\end{array}\right.$$

It is important to emphasize that even in the discrete control mode configuration, the device control commands are updated continuously, namely, a new direction command is generated and sent to the effector at every firmware runtime update (time step = 20 ms).

### Non-clinical testing

A battery of electrical and mechanical bench tests was conducted with the NuroSleeve to ensure that its specifications met the device requirements. The requirements evaluated included hand splint ROM, grasp force and speed, battery life, and the total life cycle of the splint. Our goal was to allow people to grasp objects weighing up to 1.22 kg. To accomplish this, based on the calculation presented below, our system, which has a static coefficient of friction of 0.90, requires 13,3 N of force.

$$F=m * g /\upmu$$

**Fig. 5:**
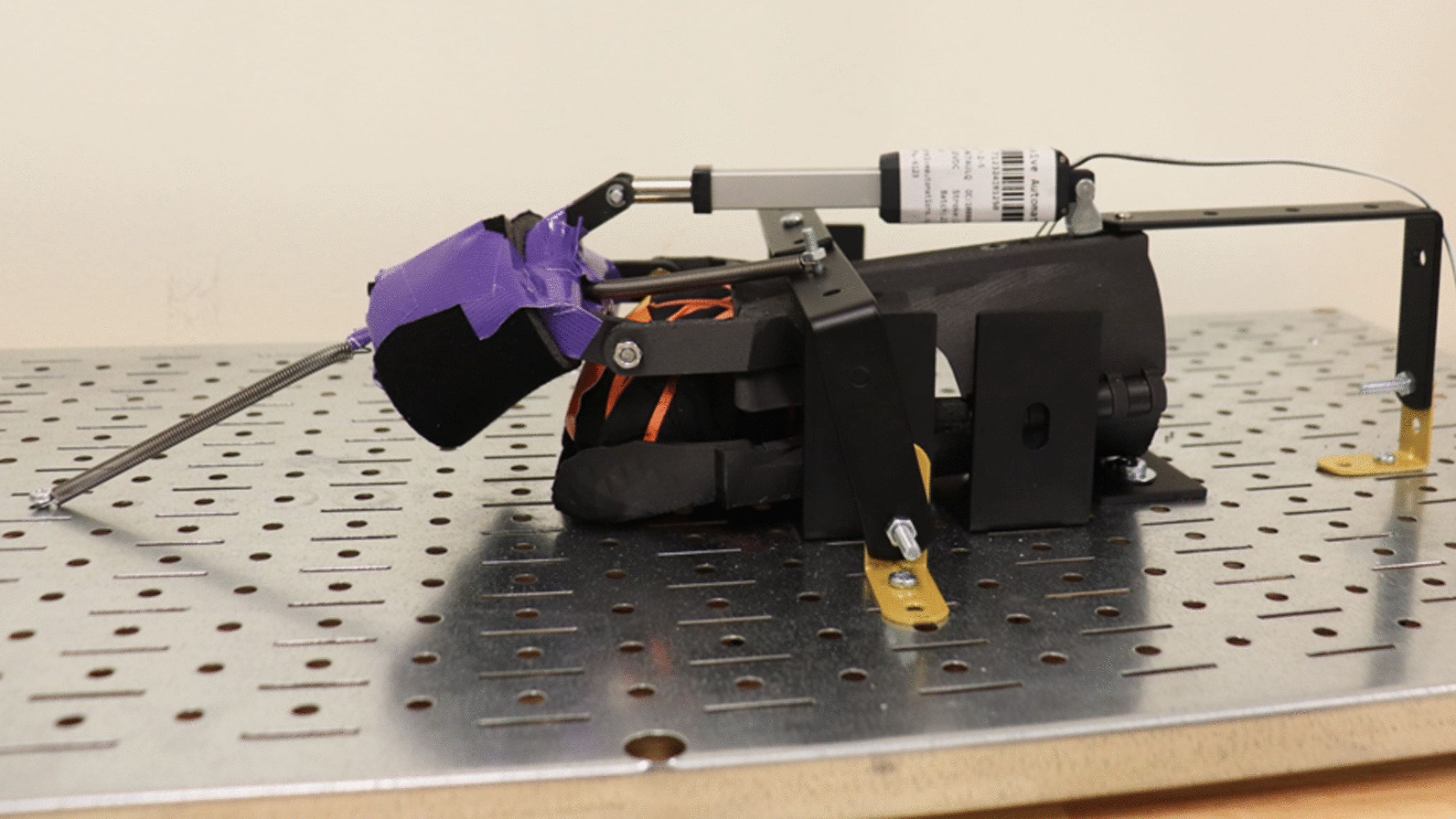
Test Bench for the NuroSleeve with added resistance with springs

In which m is the weight of the intended object to grasp, g is the gravitational acceleration, µ is the static coefficient of friction, and F is the required force. Our system is capable of producing a force of 22.4 N, but we do not wish to operate at the limit, so we conducted the experiments based on our recommendation for usage, which is 13.3 N. While manual measurements were carried out for most requirements, accelerated life testing was also implemented using two specific setups, as shown in Fig. 5. The first accelerated life test focused on the mechanical characteristics of the device, repeatedly opening and closing the hand onto a simulated test load placed below the palm to simulate object grasping. The repeated hand movements (opening/closing) were programmed to last four seconds each, while two seconds of rest were introduced between consecutive movements, resulting in a 66% linear actuator utilization (duty cycle). To fully automate the test, two force sensors and a microcontroller were integrated into the test setup to continuously monitor the device’s operation and collect data, including identifying the breaking point accurately.

The second accelerated life test deployed a similar setup with the addition of three springs connected to the splint finger piece: one for opposing hand opening and two for opposing hand closing or grasping. The springs induced a variable load with a maximum of 13.3 N in both directions. The formula for calculating the force induced by the springs is provided below:

$$\mathrm{F}={K}_{1}\left(L-\Delta x\right)-\left({K}_{2}+{K}_{3}\right)\Delta x$$

$$0<\mathrm{\Delta x}<\mathrm{L}, {\mathrm{K}}_{1}={\mathrm{K}}_{2}+{\mathrm{K}}_{3}$$

$$\mathrm{F}={K}_{1}\left(L-2\mathrm{\Delta x}\right)$$

F is the force opposing hand opening; K1 is the spring constant of the single spring opposing hand opening; K2 and K3 are the spring constants of the double springs opposing hand closing; L is the maximum possible spring enlongation; and $\Delta x$ is the spring displacement from the fully opened hand position. The single spring has the same relative displacement as the double springs, but in the opposite direction, and the spring constants are chosen such that to satisfy K_1_ = K_2_ + K_3_. The maximum net force on the linear actuator occurs when the hand is fully open or closed ($\Delta x$ = 0 or L), which is 13.3 N.

To determine the battery life of the NuroSleeve, its power consumption was analyzed. In standby or FES mode, the NuroSleeve PCB with all sensors connected consumes 175 mA of continuous current; movement of the linear actuator increases the current consumption to between 275 and 375 mA, depending on the torque generated. Assuming a nearly linear relationship between battery capacity and operating time due to the extremely low discharge current rate [75], the battery life of NuroSleeve is estimated to be between 8 and 17 h. Notably, the Chattanooga Continuum™ FES device requires two AA batteries and is not powered by NuroSleeve's primary battery. The battery life of the FES device is highly dependent on the stimulation intensity, the operational cycle, and the type of batteries used, but it is typically significantly longer than that of the NuroSleeve.

### Clinical trial

In addition to the extensive non-clinical bench electrical and mechanical testing, the NuroSleeve has been evaluated in a clinical setting by various stakeholders, including users, therapists, caregivers, and physicians. Continuous integration of stakeholder feedback is crucial to the development of a device with practical utility. The NuroSleeve is currently being evaluated in a clinical trial (NCT04798378), approved by the Thomas Jefferson University Institutional Review Board (IRB). Consenting and enrolled participants complete an 8-week rehabilitation program that incorporates the device into occupational therapy sessions and ADLs at their homes.

In brief, the trial consists of an initial (pre-intervention) clinical outcome assessment session, which establishes a baseline of each participant’s UE functional ability. The Canadian Occupational Performance Measure (COPM) [76] is also administered to identify three to five activities across multiple domains (including work, self-care, and leisure) in which the participant desires to improve their functional performance or satisfaction. Each participant then undergoes 3D scanning and receives a customized NuroSleeve, after which they engage in a series of outpatient occupational therapy sessions that incorporate the device. These one-hour sessions occur three times per week for eight weeks. During the sessions, the OT trains the participant how to don and doff the NuroSleeve splint, ensures its proper fit and function, and helps determine which components and control strategies to incorporate, based on the participant’s preferences and abilities. These sessions also involve repetitive task practice using the device as they relate to the participant’s COPM goals. These sessions were designed by the therapist for each participant individually. For example, if a participant wanted to improve their ability to cut food, the therapist would prescribe smaller repetitive tasks that would help improve their ability to achieve this using the NuroSleeve (e.g., grasping increasingly smaller items, stimulation for supination or pronation). They also practiced using the NuroSleeve to help them use their affected arm as a stabilizer for their unaffected UE during tasks.

The elements of the NuroSleeve system used during sessions vary between participants and between sessions. Personalized control strategies are selected independently based on their preferences and physical abilities. During therapy sessions, the participant and therapist can use the orthosis or FES effector independently or simultaneously. While these sessions are ongoing, if and when the participant demonstrates the ability to use the NuroSleeve independently, they take the NuroSleeve home and start to use it during daily activities. After the 8-week intervention period, the standardized outcome assessments are repeated, with and without the device being worn.

Controlling the orthosis could be achieved by joystick or IMU; control of the FES was achieved using an IMU (or a second IMU if one is being used for the motor at the same time). While EMG control was available to all participants, it has not been widely adopted because, in the early sessions with the first participant, who had extensive experience with EMG-controlled devices, it did not prove to be a reliable or consistent control method [35]. The voice recognition module was not available for the participants at the time of this paper because its development began after being requested by both NS01 and NS03, and it is undergoing testing and validation with additional participants.

## Results

Table 1 compares the main characteristics of the NuroSleeve to those of comparable commercially available UE active assistive devices. The table shows that the former can effectively combine several control methods in addition to having two potential output modalities, mechanical and electrical stimulation. The results of NuroSleeve evaluation for (1) pre-clinical bench testing and (2) clinical outcomes are detailed in the next section.

**Table 1:** Comparison between certain characteristics of comparable UE powered orthoses

Device name	FES	Motorized	EMG trigger	IMU trigger	Voice recognition trigger	Manual trigger (joystick or push button)	Continually tracks device usage	3D Printed customized	Flexibility in sensor placement	Don and Doff without assitance	Light weight (less than 1.5 kg on arm)
NuroSleeve	✓	✓	✓	✓	✓	✓	✓	✓	✓	✓	✓
MyoPro [23]	✗	✓	✓	✗	✗	✗	✗	✗	✗	✗	✗
Neomano [24]	✗	✓	✗	✗	✗	✓	✗	✗	✓	✓	✓
Power Driven Flexor Hinge [25]	✗	✓	✗	✗	✗	✓	✗	✗	✗	✓	✓
Exomotion [26]	✗	✓	✓	✗	✗	✗	✓	✓	✓	N/A	N/A
HandyRehab [27]	✗	✓	✓	✗	✗	✗	✓	✗	✓	N/A	N/A
H200 [28]	✓	✗	✗	✗	✗	✓	✗	✗	✗	N/A	✓
OmniHi5 [29]	✓	✗	✓	✗	✗	✗	✓	✗	✓	✓	✓
ReGrasp [30]	✓	✗	✗	✓	✗	✓	✗	✗	✗	✓	✓

N/A not available

### Pre-clinical bench testing

Accelerated life testing revealed that the linear actuator was the mechanical component most likely to fail. During testing in both test scenarios (with and without tension springs), the linear actuator failed before any of the 3D-printed components failed or exhibited any signs of wear and mechanical fatigue. The linear actuator failed after 86 h of continuous operation (totaling 26,361 actuations) in the first accelerated life test that simulated grasping an object and after only 47 h (totaling 14,269 actuations) in the second test under constant load with the additional 13.3 N spring force. As expected, the test with the springs caused the linear actuator to fail almost half of the time due to the increased torque requirements. In both tests, the linear actuator stopped working due to the failure of the internal gearbox, which resulted in a higher than nominal current draw of the direct current (DC) motor and eventually triggered the linear actuator’s onboard safety power cut-off circuitry. Despite the linear actuator failure preventing the NuroSleeve from being functional, such a failure mode poses no risk to the user and can therefore be categorized as a safe failure. In both accelerated life tests, the linear actuator duty cycle (ratio between the actuation and stationary time durations) was 66% (4 s on, 2 s off), which is much greater than the 10–20% nominal duty cycle specified for the PA-07 linear actuator [54]. Utilizing this linear actuator with high-duty cycles leads to excessive operational temperatures and mechanical wear, which can eventually lead to premature failure. Based on these initial findings, additional testing will be performed to better characterize the device's expected life span and maintenance intervals.

The NuroSleeve’s battery life was tested with continuous hand opening and closing movements lasting for 4 s with a 2-s rest in between. In case of the battery life, with no external loading, the battery life was 13 h; with the stated variable load of maximum 13.3 N, the battery life was 11 h. These results are better than the anticipated (calculated) battery life because the linear actuator in these tests was not operating at maximum torque. Initial clinical trial findings indicate that the NuroSleeve’s battery life in real-life usage is substantially longer, with participants only needing to recharge the battery once a week. This discrepancy is likely because the linear actuator’s “real-life” duty cycle is much lower than the one used for bench-testing and depends upon the wearer’s flexion and extension rate, body size, and level of spasticity or tone in their hand.

**Fig. 6:**
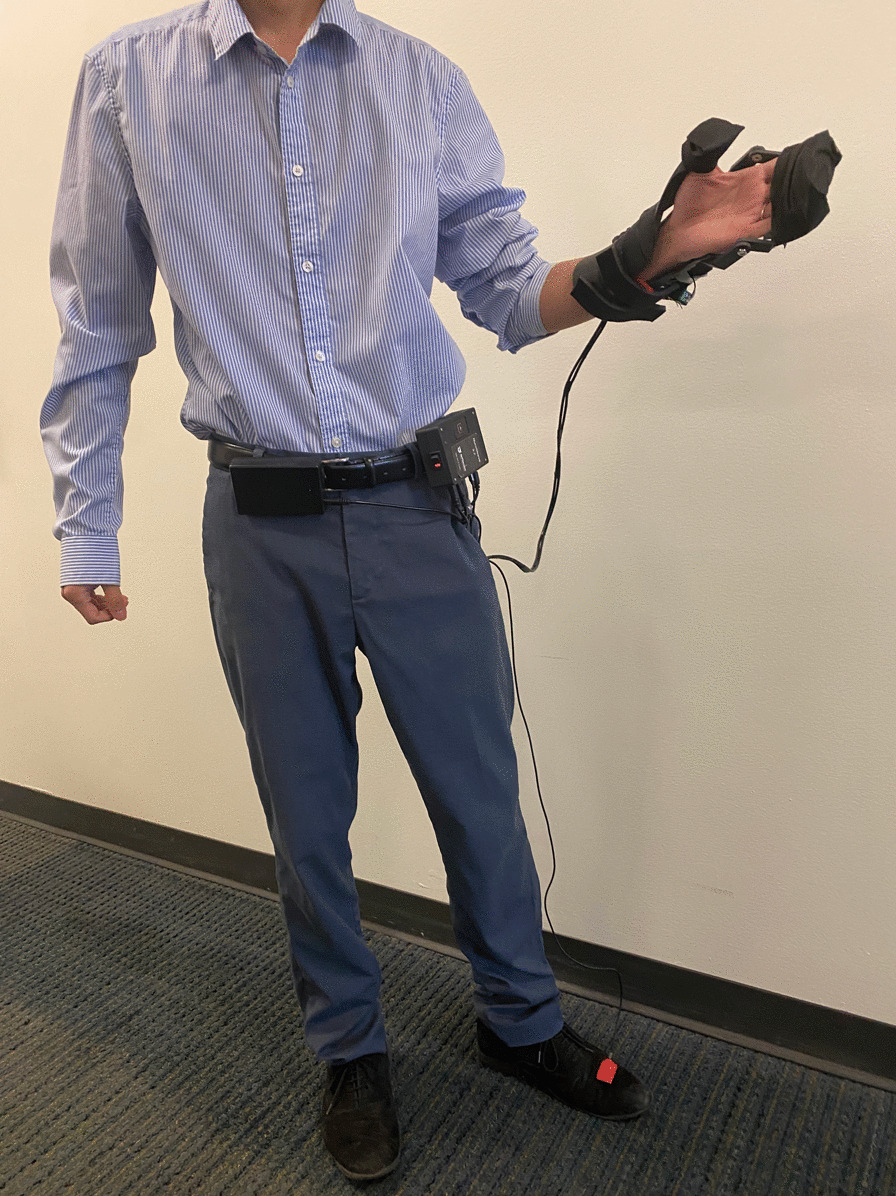
Illustration of the NuroSleeve system in operation. The MCU and battery are worn on the belt, while an IMU sensor attached to the shoe controls the splint motor in continuous mode

In order to increase user satisfaction and reduce physical problems such as pressure point formation, muscular fatigue, perspiration, and skin irritation [41], the Nurosleeve is designed to have minimal weight on the individual's arm. The only component worn on the hand is the splint, which varies in weight depending on the size of the individual's hand; for our participants, this ranged between 175 and 310 g. The control unit (114 g), battery (192 g), and FES unit (200 g) are not intended to be placed on the splint but rather in a backpack, waist bag, or wheelchair (as shown in Fig. 6). For reference, Table 2 introduces an itemized list and cost of the components, which total about $900 just for parts without including labor or the cost of facilities and equipment. The performance testing results demonstrate that the device has met the desired technical and functional requirements. The above-mentioned properties, combined with a lightweight (less than 310 g) and custom-fitting splint, ensure that the NuroSleeve device can be easily used by people with UE impairment during ADLs and can help them become more independent in both the home and community settings.

**Table 2:** The cost estimate of a NuroSleeve

Components	Estimated cost
OnyxTM filament	$61
Hardware, including nuts, bolts, Velcro, and straps	$37
Linear actuator	$70
12 V lithium-ion battery	$27
Cables	$40
Joystick	$14
IMUs	$70
EMG sensors	$60
FES	$350
PCB with components	$70
Voice recognition module	$50
Total	$849

### Clinical trial outcomes

**Fig. 7:**
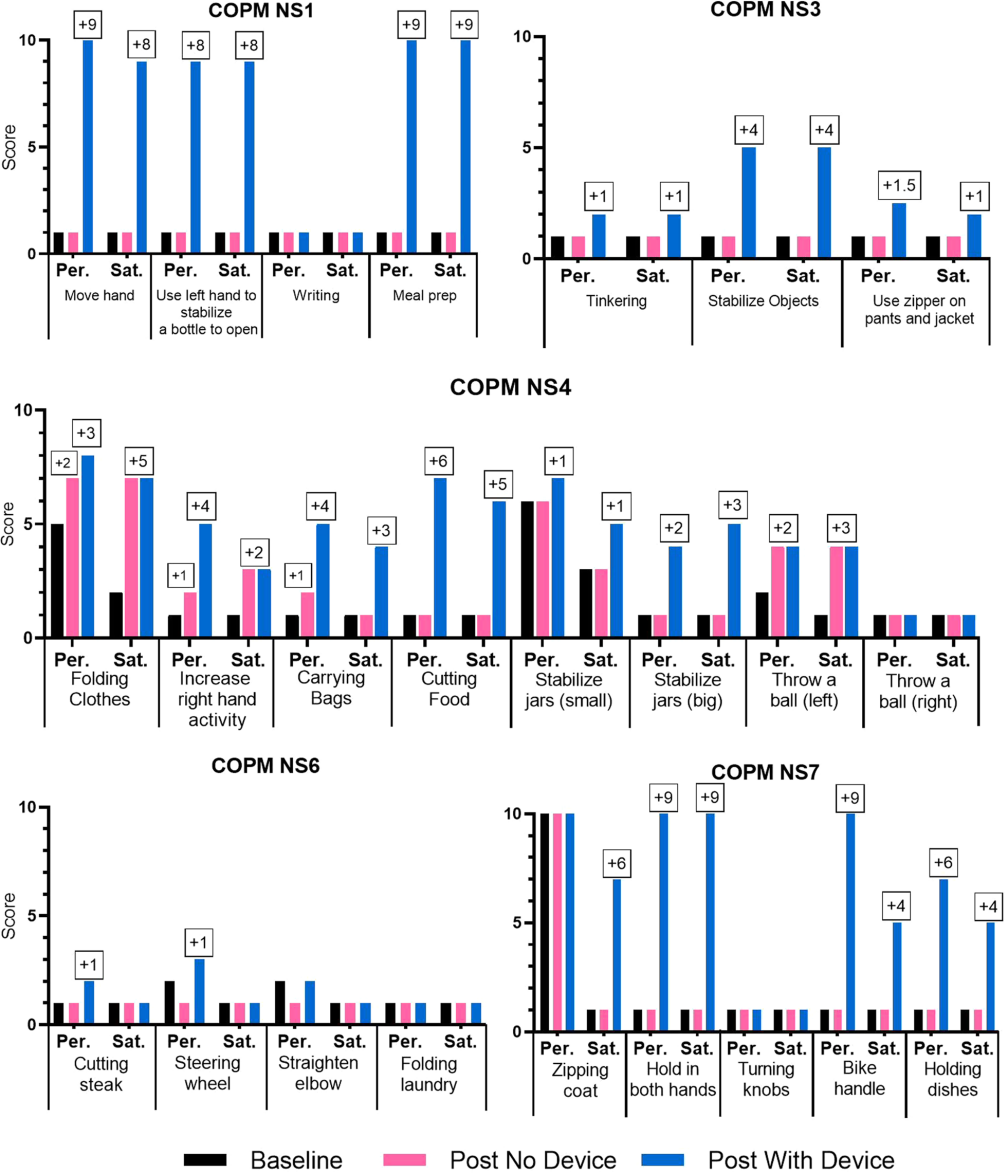
Performance and satisfaction scores on the COPM for NS1, NS3, NS4, NS6, and NS7 (Per. = Performance, Sat. = Satisfaction). COPM was performed three times, once at the beginning of the trial, and twice at the end of the trial with and without the device. The bars represent COPM scores. The black bars are for the pre-trial scores (baseline), the pink bars are for the post-trial scores without the device, and the blue bars are for the post-trial scores with the device

At the time of this report, five individuals with hemiparesis from chronic stroke had completed participation in the NuroSleeve clinical trial. The trial intervention comprises three one-hour occupational therapy sessions per week and daily home use of the NuroSleeve for a total of 8 weeks. The primary clinical outcome measure is the COPM [76], for which we report results in Fig. 7. Secondary outcome measures include the Action Research Arm Test (ARAT) [77–79], Box and Blocks Test (BBT) [19, 78], ABILHAND [80], and the Patient-Reported Outcomes Measurement Information System UE Short Form Version 2.0 (PROMIS UE SF V2.0) [81, 82]. However, the results of these will be reported in a separate paper. All outcome measures are administered both before and after the eight-week intervention. In the post-intervention evaluation, each measure is administered with and without the NuroSleeve. The COPM is a self-reported measure, so for each self-selected task, the participant rates their ability to perform it and their satisfaction with performing it. They rate it on an ordinal scale ranging from “1" (cannot do/not satisfied) to "10" (can perform well/very satisfied), both before and after the trial intervention. A two-point increase in score indicates a clinically meaningful improvement. Results from the first five trial participants (NS1, NS3, NS4, NS6, and NS7) are reported in Fig. 7. We do not have outcome data to report for participants NS2 and NS5 because, as of the time of this manuscript, they have not yet participated in therapy sessions; the study team is designing elbow and shoulder components for their respective NuroSleeves. All five participants were successfully fitted with a customized NuroSleeve and participated in all 24 occupational therapy sessions over 8 weeks. The elements of the system implemented in these sessions varied by participant and session; participants used the splint and/or FES during repetitive task practice; however, the control mechanisms used differed based upon their preferences. The five participants reported on in this manuscript received an orthosis and FES component, one or more IMU sensors, and a joystick.

The OTs were able to set up and save individual settings for each participant on the NuroSleeve, and the participants were able to select their preferred control and activation methods using the push-button on the MCU in order to readily use the orthoses. Based on 3D scans of their hands and forearms, all five participants received customized 3D-printed orthoses and used the orthosis with the joystick as the main controller because it was found to be the simplest and most reliable to use during activities. Furthermore, two participants (NS6 and NS7) used the FES effector in addition to the orthosis, and used IMU control in addition to the joystick. All participants used the joystick and/or IMUs to control NuroSleeve in their homes without requiring additional calibration or adjustments. All participants learned how to don and doff the NuroSleeve on their own and were able to use the device at home. Most importantly, all but one participant improved on the primary outcome measure and provided valuable feedback to guide future design improvements. All participants found the device to be lightweight, portable, authentic, comfortable, easy to don and doff and form-fitting, which made them willing to use it in a social setting.

The clinical findings shown in Fig. 7 demonstrate that all participants reported improvement in the performance of and satisfaction with most of their self-identified goals when the device was in use. For many participants, the improvement was clinically meaningful; in three of the four goals chosen by NS1, one of three identified by NS3, six of eight identified by NS4, and three of five identified by NS7, improvement exceeded the two-point threshold for a clinically meaningful change. NS6 did not show any clinically meaningful change. Both NS1 and NS7 reported performance and satisfaction scores of 9 while using the device, close to the maximum score of ten.

## Discussion

The integration of 3D scanning and 3D printing technologies into orthosis manufacturing has paved the way for the creation of custom, form-fitting orthoses that meet the user's anatomical and functional needs. Studies indicate that the comfort and effectiveness of 3D-printed orthoses surpass those of conventional orthoses [83]. In addition to being more comfortable and effective, the use of 3D-scanning and 3D-printing technologies can result in orthoses that are lighter in weight, authentic, and formfitting; NuroSleeve's design philosophy leverages these advantages [46, 47, 83, 84]. The design approach also improves upon the current state of the art in UE orthoses. The following paragraphs introduce a brief comparison with the state-of-the-art devices as identified in Table 1. The MyoPro [23] is a motorized arm and hand orthosis designed to help restore function in individuals with UE impairment. The MyoPro battery, MCU, and control panel are part of the orthosis itself, resulting in a total weight of approximately 1928 g, which may be uncomfortable for some individuals to wear for long periods of time. In comparison, the NuroSleeve's orthosis component includes the 3D-printed splint and possibly a joystick or IMU sensor, whose maximum weight was 310 g, based on the trial’s participants. In addition, the results indicate that the NuroSleeve can operate for approximately 11 h of continuous use, which is sufficient for a full day of use on a single charge, whereas according to the MyoPro website, it can operate for 4.5 h on a single charge. The Exomotion [26] is an emerging technology that employs 3D-scanning and 3D-printing technologies to create a form-fitting splint, and, like the Neomano [24], they have attached the battery and control unit to a location other than the splint in order to minimize the weight on the impaired arm. The HandyRehab [27] is another light-weight motorized hand splint that utilizes EMG signals to control the splint. The limitations of the above-mentioned devices derive from their control algorithms, which are either based on EMG signals or a button. The EMG signal has been shown to be challenging to use for spastic individuals owing to the contraction of antagonist muscles, cross-talk between different muscles, external noise sources, limitations with individuals suffering from paresis or the inability to produce the desired EMG signal, and sensor placement difficulties [35, 38]. However, using a push button has its own limitations, as it requires the use of a button. The Ness H200 [28], the OmniHi5 [29], and the ReGrasp [30] are three FES orthoses that, despite being lightweight, function solely by stimulating the individual's musculature and do not include a motorized orthosis to assist the individual in achieving functional movement. The OmniHi5 is designed to operate primarily based on EMG input control, whereas the ReGrasp is intended to use an IMU placed behind the individual's ears to trigger the splint, and the Ness H200 is designed to operate with a push button. The NuroSleeve is a hybrid orthosis and incorporates an electric motor and FES, which can be controlled by several methods such as voice control, EMG sensors, IMUs, and a joystick. Adding voice control and providing the OT with flexibility in IMU placement, besides having a joystick, allows the OT to have additional options to choose from as a control method based on the type and level of injury, physical ability, and the individual's personal preference in order to assist them in performing ADLs.

The current NuroSleeve is the result of several iterations based on the feedback received from study participants and OTs as part of the ongoing clinical trial. The primary design changes implemented thus far include: (1) the 3D model was altered by removing material at the wrist to avoid contact with bony prominences; (2) the thumb and cuff pieces were separated into two distinct pieces, allowing the user to more easily don and doff the splint; and (3) the joystick controller has been mounted onto the splint, making it more convenient for the user to control the device with their unimpaired hand. We found this to be extremely helpful for individuals who rely on their unaffected hand to support and move their impaired arm; since the unimpaired hand typically provides support in the proximity of the contralateral forearm, placing the joystick in this area allows for quick and easy access to it. Although the NuroSleeve can be controlled by EMG signals, which are highly correlated with voluntary UE movements, based on previous studies [38, 85–87], our experience, and participant feedback, EMG signals tend to be erratic, so this is the least preferred control method [35].

Although the clinical results presented are from a small cohort of participants in a feasibility study, it should be noted that there is no plausible physiologic or physical process by which random chance would account for the functional improvements seen in these participants; all of them had been living with chronic deficits for two or more years following a stroke and had already exhausted standard rehabilitation therapy. Most importantly, each served as their own control and compared function with and without the device, demonstrating that the benefits were due to the device and not to a non-specific mass practice effect. The functional improvements observed merit a prospective, randomized, controlled trial with an adequate sample size to be able to differentiate the relative contributions of traditional rehabilitation therapy alone, traditional rehabilitation with the NuroSleeve, and abbreviated rehabilitation with the NuroSleeve. In addition to the promising preliminary findings, this trial demonstrates a successful model for the future of UE rehabilitation methods an interdisciplinary approach where a team comprised of neurology, engineering, software development, industrial design, and occupational therapy professionals collaborates to design and create a customizable, durable, and genuinely functional assistive device.

## Conclusions

Loss of independence due to UE neuromuscular impairment represents a high socio-economic burden for society. Current rehabilitation methods and commercially available UE active orthoses seem limited in their capability to restore function and thus improve independence in individuals living with UE impairment. The NuroSleeve has been designed, developed, and clinically evaluated to address these limitations.

The NuroSleeve is an innovative user-centric 3D-printed UE active orthosis and FES system that improves function and independence in ADLs for individuals with UE neuromuscular impairment. To overcome the limitations of existing neurotechnology, the NuroSleeve deploys 3D scanning, 3D printing and introduces a design that is user-centered, lightweight, easy to don and doff, and user-friendly. Furthermore, the NuroSleeve provides innovative options for control methods (inputs) and effectors (outputs) that can be customized and used in various combinations resulting in a functionally and clinically effective active orthosis for both home and clinical use. Current control methods include manual, EMG, IMU, and voice controls, while the effectors include a linear actuator that opens and closes the hand and two channels of FES. The team will continue its research and development activities to improve upon the device’s design and functionality; for example, work is underway to incorporate an elbow brace into the NuroSleeve, which will provide the user with more degrees of freedom (DOF), particularly arm flexion and extension. We also aim to integrate wireless sensors and improve the battery life so the user may operate it for longer without needing to recharge.

The NuroSleeve was pre-clinically evaluated using state-of-the-art bench testing to ensure adequate performance, safety, and quality metrics. Bench test results demonstrate that the NuroSleeve meets all of its technical objectives and operational requirements due to its customizable and flexible hardware and firmware designs. Preliminary findings of the clinical trial reveal that NuroSleeve meets its clinical objectives and addresses previously unmet clinical needs. The NuroSleeve has shown to be beneficial to the individuals who have enrolled in the ongoing trial; however, it can only become beneficial and available to the large population of individuals living with UE impairment if it becomes a commercially available FDA-cleared medical device. To achieve this goal, a product development plan is in place, which includes an FDA-compliant Quality Management System, a redesign and optimization, further bench testing to meet all FDA requirements, and a subsequent pivotal clinical trial. Such steps will ensure that future iterations of the NuroSleeve may be available to more individuals with UE impairment and potentially become part of clinical care.

## Data Availability

All data generated or analyzed during this study are included in this published article.

## Abbreviations

3D: Three dimensional
UE: Upper extremities
SCI: Spinal cord injury
DMD: Duchenne Muscular Dystrophy
BMD: Becker Muscular Dystrophy
ADLs: Activities of Daily Living
MD: Muscular dystrophy
FES: Functional Electrical Stimulation
MCU: Main control unit
EMG: Electromyography
IMU: Inertial measurement unit
OT: Occupational therapist
CAD: Computer-aided design
MCP: Metacarpophalangeal
ROM: Range of motion
ABS: Acrylonitrile Butadiene Styrene
TENS: Transcutaneous Electrical Nerve Stimulatione
NMES: Neuromuscular Electrical Stimulation
EMI: Electromagnetic interference
PCB: Printed circuit board
Li-ion: Lithium-ion
RTM: Remote Therapeutic Monitoring
CMS: Medicare and Medicaid Services
IRB: Institutional Review Board
COPM: Canadian Occupational Performance Measure
DC: Direct current
ARAT: Action Research Arm Test
BBT: Box and Blocks Test
PROMIS UE SF V2.0: Patient-Reported Outcomes Measurement Information System UE Short Form Version 2.0
FDA: Food and Drug Administration
DOF: Degrees of freedom
